# A computational assay for identifying millet-derived compounds that antagonize the interaction between bisphenols and estrogen-related receptor gamma

**DOI:** 10.3389/fphar.2024.1435254

**Published:** 2024-10-31

**Authors:** Rajesh Kumar Pathak, Jun-Mo Kim

**Affiliations:** Department of Animal Science and Technology, Chung-Ang University, Anseong-si, Republic of Korea

**Keywords:** BPA, ERRγ, millets, virtual screening, admet, molecular dynamics simulation

## Abstract

The use of Bisphenol A (BPA) and its analogs in industries, as well as the products made from them, is becoming a significant concern for human health. Scientific studies have revealed that BPA functions as an endocrine disruptor. While some analogs of BPA (bisphenols) have been used for a longer time, it was later discovered that they are toxic, similar to BPA. Their widespread use ensures their presence in the environment, and thus, everyone is exposed to them. Scientific research has shown that BPA interacts with estrogen-related receptor gamma (ERRγ), affecting its normal function. ERRγ is involved in biological processes including energy metabolism and mitochondrial function. Therefore, continuous exposure to bisphenols increases the risk of various diseases. In our previous study, we observed that some analogs of BPA had a higher binding affinity to ERRγ compared to BPA itself and analyzed the amino acid residues involved in this interaction. We hypothesized that by antagonizing the interaction between bisphenols and ERRγ, we could neutralize their toxic effects. Taking into account the health benefits of millets and their toxin removal properties, virtual screening of millet-derived compounds was conducted along with prediction of their ADMET profiles. Top five candidates were prioritized for Density Functional Theory (DFT) calculations and further analyses. Long-term molecular dynamics simulation (1 µs) were utilized to evaluate their binding, stability, and antagonizing abilities. Furthermore, reevaluation of their binding energy was conducted using the MM-PBSA method. This study reports millet-derived compounds, namely, Tricin 7-rutinoside, Tricin 7-glucoside, Glucotricin, Kaempferol, and Setarin. These compounds are predicted to be potent competitive inhibitors that can antagonize the interactions between bisphenols and ERRγ. These compounds could potentially assist in the development of future therapeutics. They may also be considered for use as food supplements, although further investigations, including wet-lab experiments and clinical studies, are needed.

## 1 Introduction

Bisphenols, especially bisphenol A (BPA), are well-known hazardous chemicals, and their role as endocrine disruptors is well established. Additionally, several analogs of BPA are present in the environment, and these have been found to be harmful to human health. Data indicate that the annual production of BPA amounted to approximately 4.85 million tons (Eladak et al., 2015). Beginning in the 1960s, BPA found utility in the production of epoxy resins and polycarbonate plastics. These materials are utilized in the development of common items used daily, such as containers for food and drinks, toys, tooth sealants, paints, medical tools, thermal receipts, and CDs/DVDs (Huo et al., 2015; Sirasanagandla et al., 2022). Consequently, people are frequently exposed to BPA and its analogs through several routes, including oral, transdermal, and respiratory. These chemicals have been detected in urine, milk, blood, serum, plasma, hair, saliva, sweat, placenta, and amniotic fluid in different studies (Akhbarizadeh et al., 2020; Rahman et al., 2021; Tschersich et al., 2021). They are accountable for a wide range of pathological conditions in both humans and animals, including reproductive abnormalities, developmental disorders, neurobehavioral disorders, metabolic diseases, and cancers (Siracusa et al., 2018; Ma et al., 2019; Mustieles et al., 2020). Despite the adverse effects associated with BPA, its utilization in consumer products complicates efforts to reduce its prevalence and alleviate its damaging consequences. As of now, no alternative to BPA that is both safer and more cost-effective has been identified (Eladak et al., 2015; Moon, 2019).

Since the 1930s, researchers have noted that BPA can mimic the hormone estradiol. Therefore, many studies have investigated how BPA can disrupt hormone systems (Yuan et al., 2023). Estradiol is the most potent estrogen hormone and plays a crucial role in various essential physiological processes (Yasar et al., 2017). These functions include the development of reproductive organs, as well as the regulation of cardiovascular, musculoskeletal, immune, and central nervous system balance (Nelson and Bulun, 2001; Gruber et al., 2002; Yasar et al., 2017). The cellular effects of estradiol are mediated by transcription factors and the estrogen receptor (Yasar et al., 2017). Estrogen-related receptor gamma (ERRγ) is classified as an orphan nuclear receptor and functions as a constant transcription activator, playing a crucial role in regulating both mitochondrial energy generation and overall energy metabolism (Matsushima et al., 2007; Matsushima et al., 2022). The estrogenic activity of BPA is well documented, and it has been found that BPA interacts with ERRγ more strongly than the natural hormone estradiol (Babu et al., 2012). This interaction causes several dysfunctions in the normal activity of ERRγ, leading to various diseases (Delfosse et al., 2014; Tohmé et al., 2014; Gao et al., 2015). Therefore, there is a pressing need for a concerted effort to eliminate the toxic effects of bisphenols in order to maintain good health.

A previous study conducted in our lab analyzed the interaction between BPA and its analogs (bisphenols; n = 22) with ERRγ, as well as the key amino acid residues involved in these interactions (Pathak and Kim, 2024). Antagonizing these interactions can help mitigate the toxicity of bisphenols. Millets are recognized as nutri-cereal crops, commonly referred to as smart food (Lydia Pramitha et al., 2022), and they play a role in detoxification by eliminating harmful toxins and free radicals while neutralizing enzymes within organs. This contributes to the prevention of various health concerns, including cancer, diabetes, and cardiovascular diseases (Peterson et al., 2010; Rodriguez et al., 2020; Saini et al., 2021; Babele et al., 2022). Since millets are abundant sources of phytochemicals with significant nutraceutical potential, there is an urgent need for comprehensive and collaborative research dedicated to exploring the phytochemicals present in these cereals. Currently, the majority of research efforts have primarily focused on whole grains and extracts derived from millets (Kumar et al., 2021b; Shah et al., 2021). To raise awareness about the advantages of millets, the United Nations has designated 2023 as the International Year of Millets (https://www.fao.org/millets-2023/en; accessed on 7/9/2023).

**FIGURE 1:**
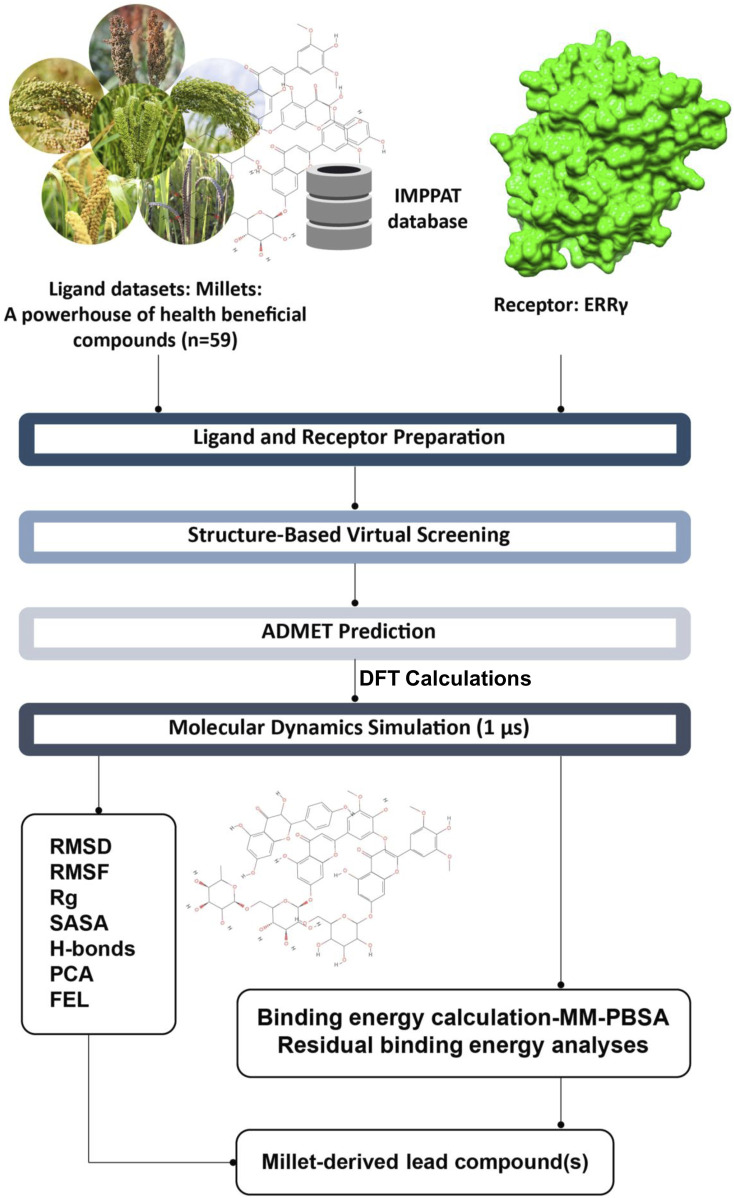
Summary of the study and the computational approaches used to identify millet-derived compounds that counteract the interactions between bisphenols and ERRγ.

Based on our previous study on the interactions of BPA and its analogs with ERRγ, as well as the importance of millets in human health, we hypothesized that phytochemicals from millets can be utilized to counteract the interactions between bisphenols and ERRγ. In this study, we conducted virtual screening of 59 millet phytochemicals targeting the interactions between BPA and ERRγ, to predict their antagonistic activity. Furthermore, we have selected the top ten millet compounds with strong binding affinity and subjected them to ADMET prediction. The top five were then prioritized for Density Functional Theory (DFT) calculations, molecular dynamics simulations, Gibbs free energy landscape analysis, and binding energy calculations using the MM-PBSA method. Through these analyses, we deciphered the mechanisms by which each of these compounds antagonize the interactions, identified the amino acid residues of ERRγ that contribute to the binding, and estimated their binding free energies. These findings will be valuable in optimizing these millet-derived compounds for further investigation and the development of therapeutics aimed at mitigating the toxicity of bisphenols (Figure 1).

## 2 Materials and methods

### 2.1 Retrieval of millet-derived compounds and the structure of estrogen-related receptor gamma

Based on information about millet-derived compounds documented in scientific literature, the 3D structures of 59 millet-derived compounds were obtained from the Indian Medicinal Plants, Phytochemistry, and Therapeutics 2.0 (IMPPAT 2.0) database (https://cb.imsc.res.in/imppat/) in protein data bank (PDB) file format (Mohanraj et al., 2018; Pathak et al., 2018; Kumar et al., 2021b; Shah et al., 2021; Kumar and Kotwal, 2023; Priya et al., 2023; Vivek-Ananth et al., 2023). OpenBabel (https://openbabel.org/wiki/Main_Page) was employed to convert the compounds into Protein Data Bank (PDB), partial charge (Q), and atom type (T) (PDBQT) files. The structure of ERRγ (PDB ID: 2E2R) was retrieved from the PDB database (https://www.rcsb.org). The protein structure was visualized and prepared with UCSF Chimera by removing all non-standard residues and co-crystallized ligand (Matsushima et al., 2022). A PDBQT file of ERRγ was then generated using AutoDock tools-1.5.6 by adding charges and polar hydrogens (https://autodock.scripps.edu/). This was followed by generating the grid box size that incorporated the amino acid residues of ERRγ interacting with bisphenols for virtual screening (Trott and Olson, 2010).

### 2.2 Structure-based virtual screening

Structure-based virtual screening is used to screen a database of small molecules against a target to identify low-energy binding modes of a ligand within the active site of a receptor (Pathak et al., 2023). This computational technique is widely employed in the identification of lead compounds in drug discovery (Singh and Pathak, 2021). In this study, we performed virtual screening of 59 millet-derived compounds, targeting BPA-ERRγ interactions using AutoDock vina (Trott and Olson, 2010). The protein-ligand complexes were generated using UCSF Chimera for visualization and analysis (Pettersen et al., 2004). Furthermore, protein-ligand interaction diagrams in both 2D and 3D were plotted using Discovery Studio Visualizer to determine the key amino acid residues contributing to the binding through various types of interactions (https://discover.3ds.com/discovery-studio-visualizer-download).

### 2.3 ADMET prediction and PAINS analysis

Absorption, Distribution, Metabolism, Excretion, and Toxicity (ADMET) properties and Pan Assay Interference Structures (PAINS) analyses of the top ten millet-derived compounds were performed using the pkCSM (https://biosig.lab.uq.edu.au/pkcsm/prediction) and SwissADME (http://www.swissadme.ch/index.php) tools. The SMILES of the selected compounds were obtained from the IMPPAT database and used as the input file for ADMET and PAINS analysis. The following were assessed: molecular properties (molecular weight, logP, rotatable bonds, hydrogen bond donor, hydrogen bond acceptor), absorption (water solubility and intestinal absorption), distribution (blood-brain barrier [BBB] permeability, central nervous system [CNS] permeability), metabolism (CYP2D6 substrate, CYP3A4 substrate, and CYP1A2 inhibitor), excretion (total clearance), toxicity (AMES toxicity and oral rat acute toxicity), and PAINS alert (Pires et al., 2015; Daina et al., 2017).

### 2.4 Density function theory (DFT) calculation

The top 5 millet-derived compounds were prioritized based on their predicted effectiveness through binding energy with ERRγ for downstream analysis. Density Functional Theory (DFT) calculations were used to evaluate the chemical reactivity of these millet compounds. Specifically, these calculations helped identify key molecular orbitals, such as the Highest Occupied Molecular Orbital (HOMO) and the Lowest Unoccupied Molecular Orbital (LUMO). This provided insights into how these millet compounds interact with ERRγ. The DFT calculations were carried out using the Jaguar-v12.2 module in Schrödinger maestro, with default settings (Schrödinger Release 2023–4; Schrödinger, LLC, New York, NY) (Bochevarov et al., 2013).

### 2.5 Molecular dynamics (MD) simulation

MD simulations were carried out using the GROMACS package, version 2018.1, with GPU acceleration (Abraham et al., 2015; Pall et al., 2020). The top five millet-derived compound-ERRγ complexes and apo-ERRγ were selected for molecular dynamics (MD) simulations. The system setup followed established protocols from previous studies (Pathak et al., 2022; Pathak et al., 2023). Ligand topology was generated using PRODRG, while protein topology was generated using pdb2gmx (Oostenbrink et al., 2004; Schuttelkopf and Van Aalten, 2004). Solvation was achieved using the simple point charge water model. To create topologies for the protein-ligand complexes, the generated protein and ligand topologies were merged. A cubic simulation box was created, and electroneutrality was ensured by adding ions. Additionally, the steepest descent minimization algorithm was employed to minimize the energy of both the protein and protein-ligand complexes. Subsequently, the system underwent equilibration through NVT and NPT simulations to maintain volume, temperature, and pressure. Finally, all systems were simulated for 1 µs. The structural stability of apo-ERRγ and millet-derived compound-ERRγ complexes was assessed through root-mean-square deviation (RMSD). The RMSD can be calculated as follows.

$$RMSD=\sqrt{\frac{1}{n}\sum {\left(r_{i}\left(t\right)-r_{i\;}ref\right)}^{2}}$$

In this equation, *n* represents the total count of atoms, *r*_*i*_ *(t)* denotes the current position of an atom at a given time *t*, and *r*_*i*_ *ref* signifies the initial positions of the atom. Essentially, *t* represents the various time points being considered.The flexibility was evaluated using the root-mean-square fluctuation (RMSF).The RMSF can be calculated as follows.

$$RMSF=\sqrt{\frac{1}{T}\sum {\left(r_{i}\left(t\right)-r_{i\;}ref\right)}^{2}}$$

Here,*T* represents the total duration of the analysis, *r*_*i*_ *(t)* denotes the current positions of an atom at a given time *t*, and *r*_*i*_ *ref* signifies the initial positions of the atom. The structural compactness was analyzed using the radius of gyration (Rg).The Rg value can be calculated as follows.

$$R_{g\;\left(z\right)}=\sqrt{\sum_{i}mi\;\left(R_{i}{\left(x\right)}^{2}+R_{i}{\left(y\right)}^{2}\right)/\sum_{i}mi}$$

In the equation, *m*_*i*_ denotes the atomic mass, *R*_*i*_ signifies various atom coordinates relative to the center of mass, and *Rg*_*(z)*_ indicates the radius of gyration around an axis (Sen Gupta et al., 2020). Furthermore, we performed protein folding and stability analysis using solvent-accessible surface area (SASA), followed by analysis of protein–ligand interactions through hydrogen bonding, and essential dynamics using principal component analysis (PCA). The GROMACS utilities ‘gmx rms’, ‘gmx rmsf’, ‘gmx gyrate’, ‘gmx sasa’, ‘gmx hbond’, ‘gmx covar’, and ‘gmx anaeig’ facilitated this analysis (https://manual.gromacs.org/documentation/2019/reference-manual/analysis.html). The 2D plotting tool Grace (https://plasma-gate.weizmann.ac.il/Grace/) was employed for graphical representation and data analysis.

### 2.6 Free energy landscape (FEL) and binding energy calculation

The analysis of the free energy landscape (FEL) was employed to evaluate the lowest energy states of ERRγ and ERRγ-millet-derived compound complexes (Pathak et al., 2023). The FEL calculations were performed using the ‘gmx sham’ utility in GROMACS. Additionally, the binding free energy of the top five millet-derived compounds complexed with ERRγ was determined using the g_mmpbsa tool, utilizing data from high-throughput molecular dynamics simulations (Kumari et al., 2014).The binding energy (G_binding_) of a protein–ligand complex can be expressed using the following equation.

$$∆G_{binding\;}=G_{complex\;}-\left(G_{protein}+G_{ligand}\right)$$

In the equation, *G*_*complex*_ represents the overall binding free energy of the complex, *G*_*protein*_ corresponds to the receptor in its unbound state, and *G*_*ligand*_ represents the ligand in its unbound form. The calculation of the energy contribution of amino acid residue ‘x' involved in an interaction was performed as follows.

$$∆R_{x}^{BE}=\sum_{i=0}^{n}\left(A_{i}^{bound}-A_{i}^{free}\right),$$

In this equation, *n* represents the total residues count, while *A*_*i*_^*bound*^ and *A*_*i*_^*free*^ represent the bound and free energy of the *i* ^*th*^ atom within each *x* residue, respectively.

## 3 Results

### 3.1 Predicting millet-derived compounds that inhibit the interaction between bisphenols and ERRγ

Structure-based virtual screening can be employed to identify the most favorable interactions between a receptor and ligand by utilizing a chemical compound database. This method is valuable for evaluating which ligand would effectively interact with a target to form a complex. The ligands are then ranked based on their binding free energy with the target. In this study, we employed 59 compounds derived from millet to conduct virtual screening against the interaction of Bisphenols with ERRγ, in search of potential hits. We assessed the binding free energy of each compound to identify these potential hits for further assessment. Typically, a protein-ligand complex with a low binding energy indicates a strong binding affinity. Therefore, we selected the top 10 screened compounds with the lowest binding energies, ranging from −8.5 to −7.7 kcal/mol, as potential lead compounds. The names of the millet-derived compounds, their IMPPAT and PubChem IDs, binding energy, type of interaction, and interacting amino acid residues are listed in Table 1. The binding free energies of all selected compounds with ERRγ are listed in Supplementary Table S1.

**TABLE 1:** List of the top 10 screened millet-derived compounds, with their IMPPAT/PubChem ID, binding free energies, types of bonding interactions, and interacting amino acid residues of ERRγ.

S.N.	Millets-derived compounds	IMPPAT/PubChem ID	Binding energy (kcal/mol)	Type of interaction	Interacting residues
1	Tricin 7-rutinoside	IMPHY013418/CID:44258273	−8.5	Conventional hydrogen bond, carbon hydrogen bond, pi-cation, pi-anion, alkyl, pi-alkyl	Glu245, Lys248, Ile249, Arg316
2	Tricin 7-glucoside	IMPHY013146/CID:5322022	−8.4	Conventional hydrogen bond, carbon hydrogen bond, pi-anion, alkyl, pi-alkyl	Glu245, Pro246, Lys248, Gly312, Tyr315, Arg316, Ser319, Phe320, Val325, Asp328, Lys363
3	Glucotricin	IMPHY015099/CID:13984469	−8.2	Conventional hydrogen bond, carbon hydrogen bond, pi-cation, pi-anion, alkyl, pi-alkyl	Glu245, Lys248, Ile249, Glu275, Arg316, Leu318, Ser319, Phe366
4	Kaempferol	IMPHY004388/CID:5280863	−7.8	Conventional hydrogen bond, pi-cation, pi-anion, pi-alkyl	Glu245, Pro246, Lys248, Glu275, Arg316, Lys363, Lys370
5	Setarin	IMPHY010802/CID: 131752308	−7.8	Pi-sigma, pi-pi t-shaped, alkyl, pi-alkyl	Leu268, Ala272, Leu309, Val313, Tyr326, Leu345, Ala431, Phe435
6	beta-Amyrin	IMPHY012223/CID:73145	−7.8	Pi-sigma, alkyl	Tyr315, Leu318
7	Isoorientin	IMPHY001801/CID:114776	−7.7	Conventional hydrogen bond, carbon hydrogen bond, unfavorable donor-donor, unfavorable acceptor-acceptor,pi-cation, pi-anion, pi-donor hydrogen bond, pi-pi t-shaped, pi-alkyl	Glu245, Pro246, Lys248, Ile249, Glu275, Tyr315, Arg316, Lys363
8	Tricin	IMPHY005601/CID:5281702	−7.7	Conventional hydrogen bond, carbon hydrogen bond, unfavorable donor-donor, pi-cation, pi-anion, alkyl, pi-alkyl	Pro246, Lys248, Tyr315, Arg316, Lys370, Glu275, Lys363
9	Cryptochlorogenic acid	IMPHY007451/CID:9798666	−7.7	Conventional hydrogen bond, carbon hydrogen bond, pi-alkyl interaction	Glu245, Pro246, Glu247, Tyr315, Arg316, Lys370, Lys363
10	Luteolin 7-rutinoside	IMPHY008873/CID:44258082	−7.7	Conventional hydrogen bond, Unfavorable acceptor-acceptor, pi-anion, pi-alkyl	Pro253, Asp259, Ser260, Ala264, Ile331, Gln336

### 3.2 Structural visualization and interaction analysis

**FIGURE 2:**
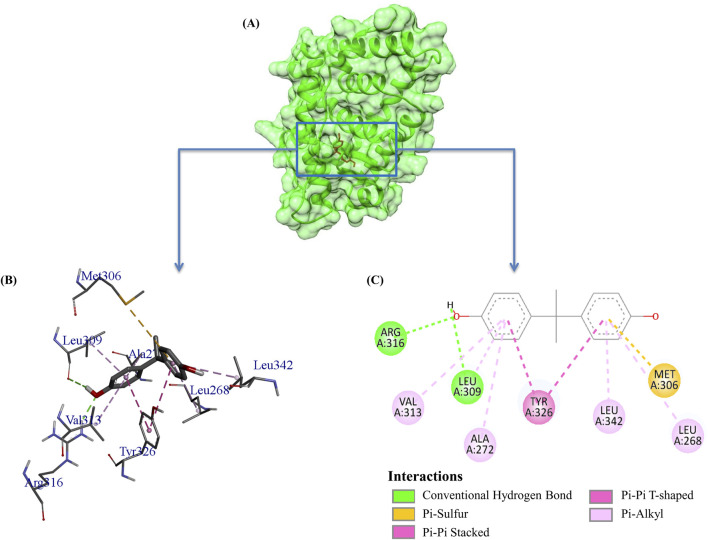
Visualization of the interaction between Bisphenol A (BPA) and ERRγ, highlighting key amino acid residues involved in the interactions. The complex was obtained by redocking the co-crystallized ligand BPA with its receptor ERRγ from our previous study. **(A)** Surface view; **(B)** 3D view; and **(C)** 2D view illustrate different types of bonding interactions.

**FIGURE 3:**
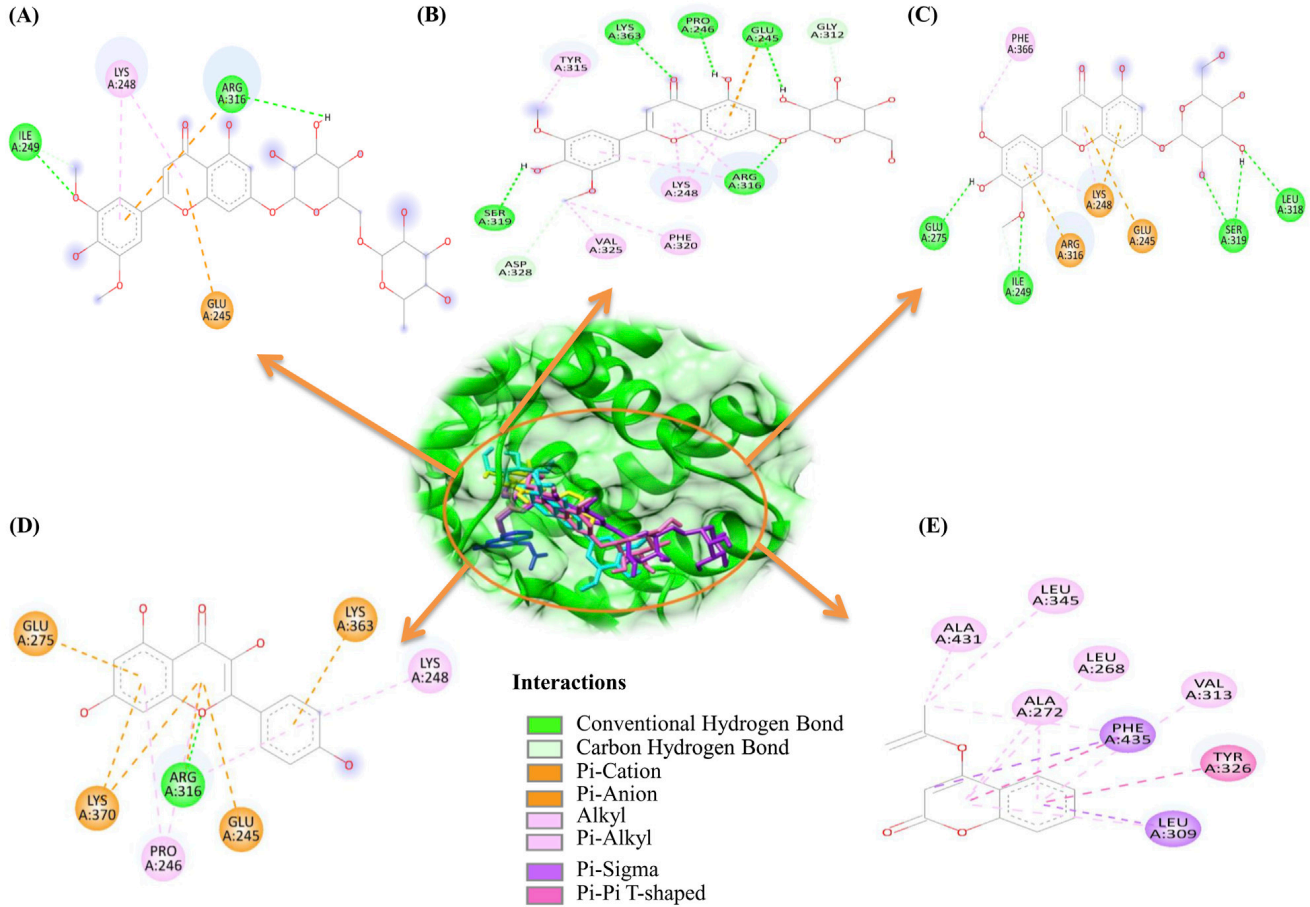
2D representations of the binding interactions of the top five millet-derived compounds at the site where bisphenols bind to ERRγ. The obtained positions of these top-interacting compounds are shown within the ERRγ binding pocket. These figures illustrate key amino acid residues that contribute to the protein–ligand interactions for antagonizing bisphenol binding. **(A)** ERRγ–Tricin 7-rutinoside. **(B)** ERRγ–Tricin 7-glucoside. **(C)** ERRγ–Glucotricin. **(D)** ERRγ–Kaempferol. **(E)** ERRγ–Setarin.

Structural visualization and interaction analysis play a significant role in investigating the interaction of amino acid residues in the target and types of bonding interactions with the ligand (Figure 2). The interaction analysis of the top ten millet-derived compounds was conducted. Tricin 7-rutinoside is one of the promising candidates that interacted with the ERRγ amino acid residues Ile249 and Arg316 through conventional hydrogen bonds. Ile249 also formed a carbon hydrogen bond, and Arg316 interacted with a pi-cation interaction; Lys248 interacted with alkyl and pi-alkyl interaction; Glu245 interacted with a pi-anion interaction. The binding energy of the Tricin 7-rutinoside with the bisphenols–ERRγ interaction was predicted to be −8.5 kcal/mol. Tricin 7-glucoside interacted with Glu245, Pro246, Arg316, Ser319, and Lys363 through conventional hydrogen bonds. Glu245 and Arg316 also interacted with pi-anion and alkyl/pi-alkyl interactions, respectively; Gly312 and Asp328 formed carbon hydrogen bonds; Lys248 formed an alkyl and pi-alkyl interaction; Tyr315, Phe320, and Val325 interacted with alkyl interactions. The binding energy of Tricin 7-glucoside was predicted to be −8.4 kcal/mol. Glucotricin interacted with Glu275, Ile249, Leu318, and Ser319 through conventional hydrogen bonds; Ser319 formed two conventional bonds, and Ile249 formed one conventional and one carbon hydrogen bond. The other mentioned amino acid residues formed single conventional bonds. Lys248 interacted with alkyl, pi-alkyl, and pi-cation interactions; Glu245 and Arg316 interacted with pi-cation and pi-anion bonding; Phe366 interacted through alkyl interaction. The binding energy of Glucotricin was predicted to be −8.2 kcal/mol. Kaempferol interacted with Arg316 through conventional hydrogen bonds, pi-anion, and pi-alkyl interaction. Pro246 and Lys248 interacted with pi-alkyl interaction; Glu245 interacted with pi-anion bonding; Glu275 and Lys363 interacted with pi-anion bonding; Lys370 interacted with pi-cation and pi-anion bonding. The binding energy of Kaempferol was predicted to be −7.8 kcal/mol. Setarin interacted with Leu345 and Ala431 through alkyl interaction; Leu268, Ala272, and Val313 interacted with pi-alkyl interaction; Tyr326 interacted with pi-pi t-shaped bonding; Leu309 interacted with pi-sigma and pi-alkyl interaction; Phe435 interacted with pi-pi t-shaped and pi-sigma bonding. The binding energy of Setarin was predicted to be −7.8 kcal/mol (Figure 3). The binding energy, type of interaction, and interacting amino acid residues of the remaining selected compounds, namelybeta-Amyrin, Isoorientin, Tricin, Cryptochlorogenic acid, and Luteolin 7-rutinoside, are provided in Table 1. Additionally, the binding interactions of bisphenol A (BPA) and the millet-derived compounds Tricin 7-rutinoside and Setarin with ERRγ are shown in Supplementary Figure S1.

### 3.3 Assessment of physicochemical properties and toxicity prediction

The assessment of physicochemical properties and prediction of toxicity help in predicting drug-like compounds, significantly reducing the chances of failures in later stages. In this study, pkCSM and SwissADME were used to predict the Absorption, Distribution, Metabolism, Excretion, and Toxicity (ADMET) properties. The predicted molecular weight of the top screened millet-derived compounds ranged from 202.209 to 638.575 Da. Other parameters were as follows: logP, −1.3927 to 8.1689; rotatable bonds, 0 to 8; hydrogen bond acceptor, 1 to 16; hydrogen bond donor, 0 to 9; topological polar surface area (TPSA), 20.23 to 249.2 Å^2^. Absorption parameters: water solubility, −6.531 to −2.428; intestinal absorption 20.029 to 95.625. Distribution parameters: BBB permeability, −1.979 to 0.667; CNS permeability, −4.957 to −1.773. Metabolism parameters: CYP2D6 substrate, “No” for all compounds; CYP3A4 substrate, “No” for all compounds; and CYP1A2 inhibitor, “Yes” for Tricin and “No” for all other compounds. The total clearance was predicted in the range of −0.226 to 0.875 for excretion evaluation. In terms of toxicity, none of the compounds were predicted to have AMES toxicity. The values of Oral Rat Acute Toxicity and Oral Rat Chronic Toxicity ranged from 2.073 to 2.577 and from 0.873 to 5.208, respectively. In addition, none of the compounds were found to have Pan Assay Interference Structures (PAINS) except for Isoorientin, Cryptochlorogenic acid, and Luteolin 7-rutinoside, which were predicted to have a PAINS alert for catechol_A. The computed values for each compound are provided in Table 2.

**TABLE 2:** Predicted ADMET properties of the top ten millet-derived compounds.

Property	Parameter	Tricin 7-rutinoside	Tricin 7-glucoside	Glucotricin	Kaempferol	Setarin	Beta-amyrin	Isoorientin	Tricin	Cryptochlorogenic acid	Luteolin 7-rutinoside
Molecular properties	MW	638.575	492.433	492.433	286.239	202.209	426.729	448.38	330.292	354.311	594.522
	LogP	−1.0811	0.0671	0.0671	2.2824	2.7054	8.1689	−0.2027	2.594	−0.6459	−1.3927
	Rotatable bonds	8	6	6	1	2	0	3	3	4	6
	HB acceptor	16	12	12	6	3	1	11	7	8	15
	HB donor	8	6	6	4	0	1	8	3	6	9
	TPSA (Å^2^)	247.43	188.51	188.51	111.13	39.44	20.23	201.28	109.36	164.75	249.20
Absorption	Water solubility (log mol/L)	−2.924	−2.857	−2.857	−3.04	−2.577	−6.531	−2.9	−3.276	−2.428	−2.904
	Intestinal absorption (% Absorbed)	28.733	43.908	43.908	74.29	95.625	93.733	61.768	89.713	20.029	25.033
Distribution	BBB permeability (log BB)	−1.916	−1.827	−1.827	−0.939	0.227	0.667	−1.564	−1.115	−1.593	−1.979
	CNS permeability (log PS)	−4.957	−4.427	−4.427	−2.228	−2.402	−1.773	−3.939	−3.411	−3.791	−4.889
Metabolism	CYP2D6 substrate (Yes/No)	No	No	No	No	No	No	No	No	No	No
	CYP3A4 substrate (Yes/No)	No	No	No	No	No	No	No	No	No	No
	CYP1A2 inhibitor (Yes/No)	No	No	No	No	No	No	No	Yes	No	No
Excretion	Total Clearance (log mL/min/kg)	−0.132	0.602	0.602	0.477	0.875	−0.044	0.372	0.62	0.298	−0.226
Toxicity	AMES toxicity (Yes/No)	No	No	No	No	No	No	No	No	No	No
	Oral Rat Acute Toxicity (LD50) (mol/kg)	2.506	2.577	2.577	2.449	2.226	2.478	2.55	2.229	2.073	2.515
	Oral Rat Chronic Toxicity (LOAEL) (log mg/kg_bw/day)	3.123	4.332	4.332	2.505	1.933	0.873	5.208	1.82	3.463	3.491
Pan Assay Interference Structures	PAINS alert	0	0	0	0	0	0	1 alert: catechol_A	0	1 alert: catechol_A	1 alert: catechol_A

### 3.4 Exploring molecular properties using DFT calculations

The frontier molecular orbitals, specifically the HOMO and LUMO, of the top five millet-derived compounds selected based on their binding free energy with ERRγ were analyzed using DFT calculations. The analysis was performed using the Jaguar module in Schrödinger Maestro. This method provides insight into the energy levels of the occupied and unoccupied orbitals. Supplementary Figure S2 illustrates the frontier molecular orbitals of the selected compounds. A larger energy gap typically indicates higher chemical stability and lower reactivity, while a smaller energy gap suggests reduced stability and increased reactivity due to easier electron transitions. According to Table 3 the calculated energy gaps range from 3.80 to 4.78 eV. Among the compounds analyzed, Tricin 7-rutinoside, Tricin 7-glucoside, Glucotricin, and Setarin exhibit the largest energy gaps, indicating higher stability. Kaempferol has an energy gap of 3.80 eV, which is lower than the other selected compounds. This suggests that the chemical reactivity of these millet-derived compounds is comparable, allowing favorable interactions with ERRγ. The detailed results are presented in Table 3.

**TABLE 3:** Comparison of energy levels for the Highest Occupied Molecular Orbital (HOMO), Lowest Unoccupied Molecular Orbital (LUMO), and the energy gap of compounds from millets.

Compound	HOMO (eV)	LUMO (eV)	HOMO-LUMO energy gap (eV)
Tricin 7-rutinoside	−5.41	−1.30	4.10
Tricin 7-glucoside	−5.40	−1.26	4.13
Glucotricin	−5.42	−1.30	4.11
Kaempferol	−5.29	−1.48	3.80
Setarin	−6.33	−1.55	4.78

### 3.5 Assessing the structural dynamics of ERRγ using MD simulation in both unbound and bound states

In recent years, the significance of MD simulation in drug discovery has greatly increased. These simulations provide in-depth information on the atomic-level behavior of proteins and other biomolecular systems. To evaluate the structural dynamics of ERRγ in both unbound and bound states, MD simulations were carried out for 1 µs. Various parameters were utilized to provide a comprehensive overview of the results, including measures such as root mean square deviation (RMSD), root mean square fluctuation (RMSF), radius of gyration (Rg), solvent accessible surface area (SASA), hydrogen bonding interactions (H-bond), essential dynamics, *i.e.*, principal component analysis (PCA), and free energy landscape (FEL) analysis.

#### 3.5.1 Conformational stability

**FIGURE 4:**
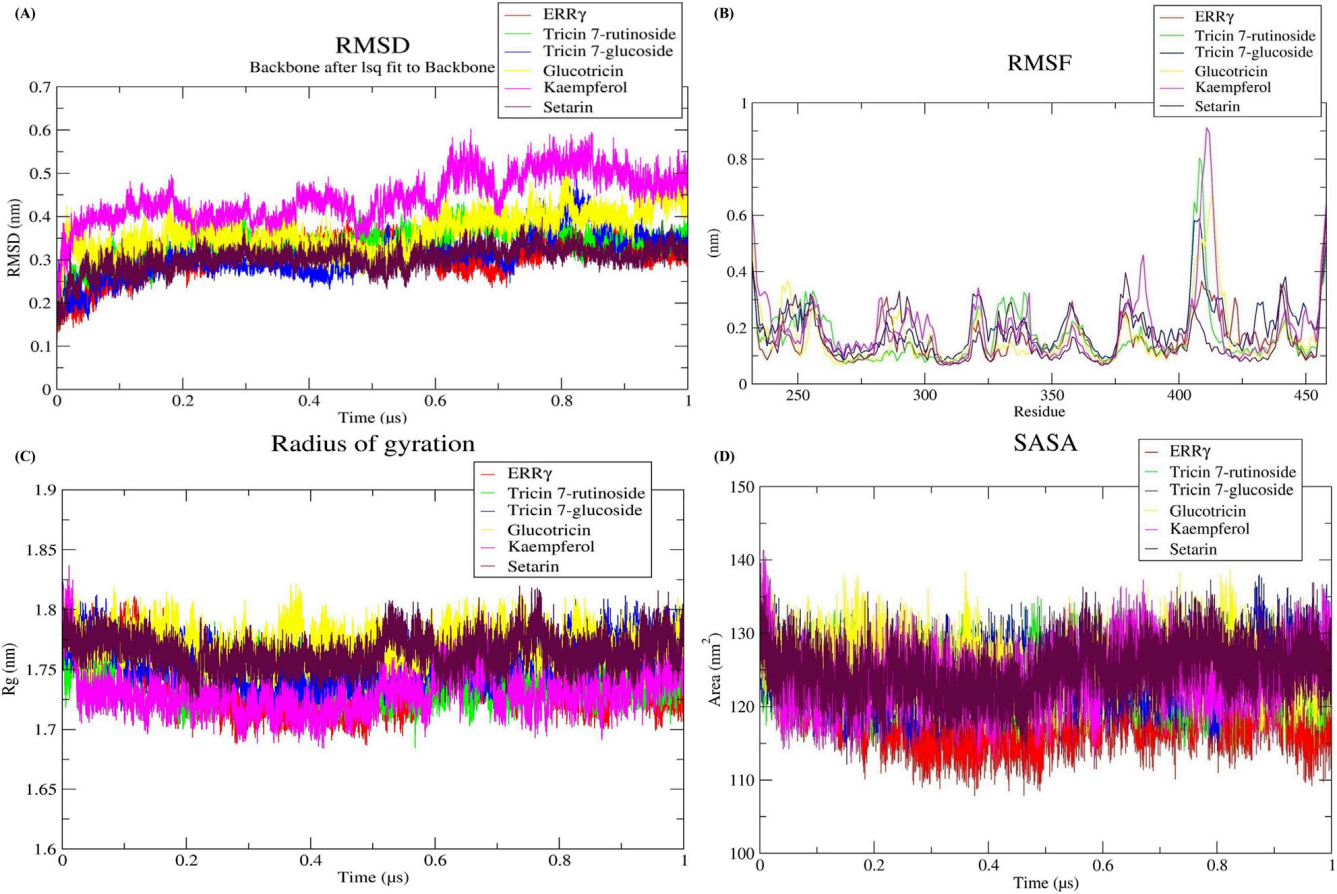
**(A)** Stability analysis using root mean square deviation (RMSD). **(B)** Flexibility analysis using root mean square fluctuation (RMSF). **(C)** Compactness analysis using radius of gyration (Rg). **(D)** Solvent accessible surface area analysis using SASA values for ERRγ–millet-derived compound complexes during a 1 µs simulation.

We assessed the conformational stability of ERRγ through RMSD analysis of a molecular dynamics simulation trajectory. A lower RMSD value indicates a higher degree of conformational stability. We analyzed the RMSD over a duration of 1 µs to gain insights into conformational changes during that time period. The ERRγ protein and all selected docked complexes showed lower RMSD values in the analysis of backbone C-alpha atoms, with the RMSD values of the docked complexes being close to the average RMSD of ERRγ. The average RMSD of ERRγ was calculated as 0.30 nm. However, the RMSD values of the ERRγ-Tricin 7-rutinoside, ERRγ-Tricin 7-glucoside, ERRγ-Glucotricin, ERRγ-Kaempferol, and ERRγ-Setarin complexes were 0.33, 0.30, 0.36, 0.44, and 0.30 nm, respectively. During the simulation, it was observed that all systems reached a stable state (Figure 4A).

#### 3.5.2 Flexibility and residual mobility

We assessed the flexibility and residual mobility of ERRγ to understand the behavior of protein after ligand binding. For this purpose, we conducted RMSF analyses. The average RMSF value for ERRγ was determined to be 0.16 nm. However, the RMSF values for the ERRγ-Tricin 7-rutinoside, ERRγ-Tricin 7-glucoside, ERRγ-Glucotricin, ERRγ-Kaempferol, and ERRγ-Setarin complexes were found to be 0.17, 0.20, 0.16, 0.20, and 0.15 nm, respectively (Figure 4B).

#### 3.5.3 Compactness

To understand the variation in stability and folding of the protein structure over time, the Rg value can be used as an assessment tool. These values provide information about the compactness of the protein structure. We calculated the Rg values for ERRγ and its complexes. The average Rg value for ERRγ was determined to be 1.73 nm. However, the Rg values for the ERRγ-Tricin 7-rutinoside, ERRγ-Tricin 7-glucoside, ERRγ-Glucotricin, ERRγ-Kaempferol, and ERRγ-Setarin complexes were found to be 1.74, 1.75, 1.77, 1.73, and 1.76 nm, respectively (Figure 4C).

#### 3.5.4 Solvent accessible surface area (SASA)

The SASA values were calculated during the entire simulation period to assess the effect of ligand on the solvent accessible surface area. The average SASA value for ERRγ was determined to be 118.59 nm^2^. However, the SASA values for the ERRγ-Tricin 7-rutinoside, ERRγ-Tricin 7-glucoside, ERRγ-Glucotricin, ERRγ-Kaempferol, and ERRγ-Setarin complexes were found to be 123.89, 124.41, 125.49, 123.83, and 124.79 nm^2^, respectively (Figure 4D).

#### 3.5.5 Hydrogen bond-based interaction study

Hydrogen bonds (H-bonds) play a crucial role in stabilizing the interactions between a protein and a ligand. To thoroughly evaluate these interactions, we performed a comprehensive analysis of hydrogen bonding during the simulation. The ERRγ-Tricin 7-rutinoside complex formed 0–4 H-bonds. However, the ERRγ-Tricin 7-glucoside, ERRγ-Glucotricin, ERRγ-Kaempferol, and ERRγ-Setarin complexes formed 0–5, 0–4, 0–5, and 0–2 H-bonds, respectively. This observation suggests that there is consistent stability in the interactions between these millet compounds and the interaction site of bisphenols-ERRγ (Supplementary Figure S3).

#### 3.5.6 Essential dynamics

We conducted a principal component analysis (PCA) to understand the essential dynamics and significant structural changes that occur when a ligand binds to the protein. In general, the initial eigenvectors play a crucial role in governing the overall motion of the protein. Therefore, we utilized the first 50 eigenvectors to study alterations in structural dynamics. To gain a more precise understanding of the movements induced by ligand binding, we computed the proportions of correlated motions from the first ten eigenvectors. The first ten eigenvectors accounted for 76.93% of the motions for ERRγ. However, the ERRγ-Tricin 7-rutinoside, ERRγ-Tricin 7-glucoside, ERRγ-Glucotricin, ERRγ-Kaempferol, and ERRγ-Setarin complexes showed 80.94%, 79.26%, 79.03%, 83.76%, and 77.30% correlated motions, respectively. Here, we observed that the ERRγ-Setarin, ERRγ-Glucotricin, and ERRγ-Tricin 7-glucoside complexes showed lower values compared to ERRγ-Tricin 7-rutinoside (Supplementary Figure S4A). Additionally, the 2D plot of these complexes was also analyzed. The initial eigenvectors of the protein reveal its core dynamics. Consequently, we examined the first two of these eigenvectors in a phase space, which depicted a stable cluster for all the selected complexes (Supplementary Figure S4B).

#### 3.5.7 Gibbs free energy landscape (FEL)

**FIGURE 5:**
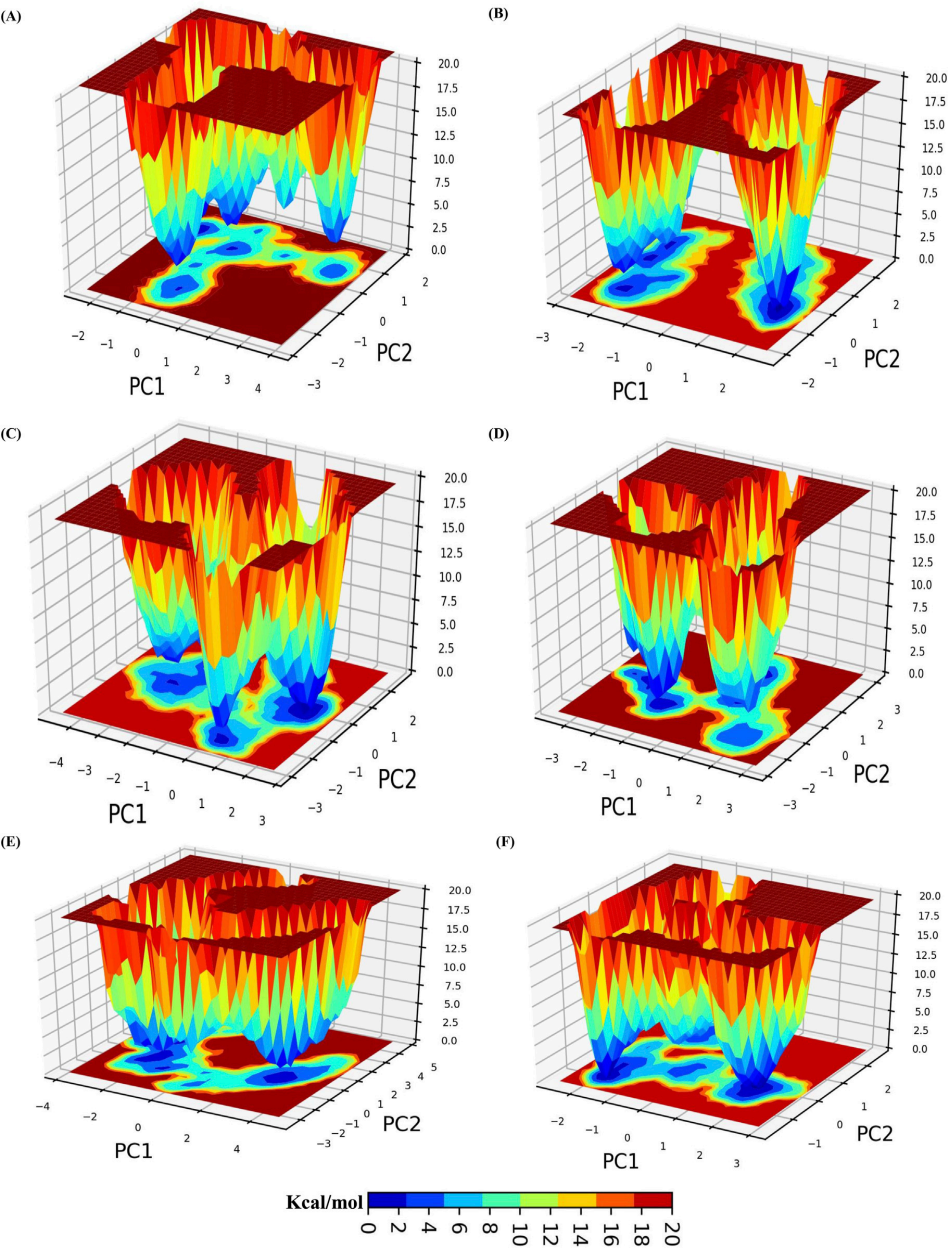
A color-coded representation of the Gibbs free energy landscape (FEL) for ERRγ and ERRγ–millet-derived compounds complexes, generated using the principal components PC1 and PC2. The color scale indicates the Gibbs free energies (kcal/mol) for different conformational states. Blue represents the lowest energy states, while red represents the highest energy states. **(A)** ERRγ. **(B)** ERRγ-Tricin 7-rutinoside. **(C)** ERRγ-Tricin 7-glucoside. **(D)** ERRγ-Glucotricin. **(E)** ERRγ-Kaempferol. **(F)** ERRγ-Setarin.

We utilized the initial two principal components to calculate the Gibbs free energy landscape (FEL). The FEL for each system is depicted in Figure 5. In the plot, the deep blue color represents the conformation with the lowest energy, whereas the red color indicates the conformation with the highest energy. The deep blue valleys represent transition states with lower energy. As observed, ERRγ has energy minima enriched with blue color and a wide space. The ERRγ-Tricin 7-rutinosidecomplex showed the deepest minima with two lowest energy funnels, indicating that it is energetically more stable than ERRγ and undergoes less frequent changes in conformational states. Similarly, the ERRγ-Tricin 7-glucoside complex had the deepest minima with three funnels. However, the ERRγ-Glucotricin and ERRγ-Kaempferol complexes spanned a large area of conformational space but still had the deepest minima. The ERRγ-Setarin complex showed two energy funnels connected to each other, covering a larger blue area. Overall, the FEL analysis indicated that all the systems exhibit low energy conformations after ligand binding and form stable complexes (Figure 5).

#### 3.5.8 Binding free energy calculation

We employed the MM-PBSA method to assess the binding free energy of all simulated complexes, thereby confirming their affinities towards bisphenols-ERRγ interactions. The binding free energies were calculated based on the final 10 ns of the MD simulation trajectories. The binding free energy for ERRγ-Tricin 7-rutinoside, ERRγ-Tricin 7-glucoside, ERRγ-Glucotricin, ERRγ-Kaempferol, and ERRγ-Setarin complexes were found to be −134.843, −173.650, −203.530, −113.351, and −124.159 kJ mol^-1^, respectively. The values for van der Waals, electrostatic, polar solvation, SASA, and binding free energies are provided in Table 4.

**TABLE 4:** Binding energies (kJ/mol) of the top five compounds obtained from millets with ERRγ as determined through molecular dynamics simulations trajectories calculated using the MM-PBSA method (van der Waals and electrostatic forces, polar solvation, SASA, and binding free energy).

Compound name	van der waals energy	Electrostatic energy	Polar solvation energy	SASA energy	Binding energy
Tricin 7-rutinoside	−161.276 ± 10.755	−11.582 ± 6.233	54.578 ± 16.471	−16.563 ± 1.471	−134.843 ± 13.750
Tricin 7-glucoside	−236.689 ± 11.295	−12.294 ± 7.179	98.025 ± 16.566	−22.692 ± 1.289	−173.650 ± 14.997
Glucotricin	−259.442 ± 11.656	−15.196 ± 10.182	94.496 ± 25.943	−23.388 ± 1.198	−203.530 ± 21.031
Kaempferol	−156.996 ± 11.924	−11.403 ± 6.923	71.041 ± 20.050	−15.992 ± 1.070	−113.351 ± 19.096
Setarin	−137.668 ± 7.205	−7.725 ± 3.414	34.946 ± 5.423	−13.712 ± 0.814	−124.159 ± 7.413

**FIGURE 6:**
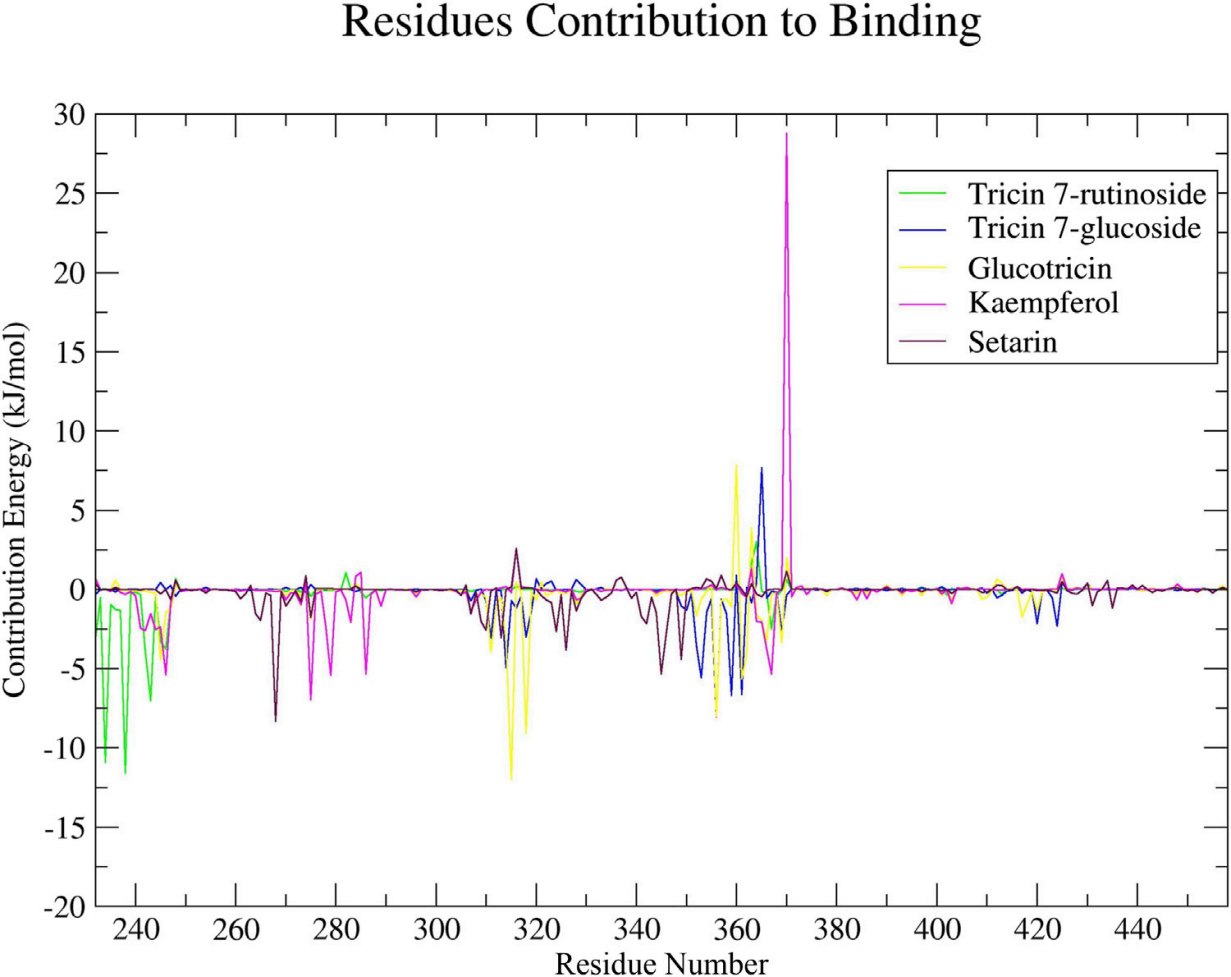
The decomposition of residual binding free energy provides valuable insights into the specific contributions of individual amino acid residues of ERRγ to the binding of millet-derived compounds.

To identify the amino acid residues that are crucial for ligand binding, we conducted residual binding energy analyses on the simulated complexes. All of the selected compounds exhibited significant involvement in interactions with amino acid residues of ERRγ, which are associated with interactions with bisphenols, suggesting that these compounds have the potential to act as antagonists for these interactions. Amino acid residues within positions 232 to 245, 260 to 290, and 310 to 375 made significant contributions to these interactions (Figure 6).

## 4 Discussion

Bisphenol A (BPA) and its analogs are commonly used chemicals in the production of polycarbonate plastics and epoxy resins. These industrial compounds have the ability to mimic estrogen, thereby disrupting the endocrine system (Babu et al., 2012; Nayak et al., 2022; Kodila et al., 2023). BPA is so prevalent that it can be found everywhere. One of the primary ways it enters the human body is through the consumption of water, food, and drinks that have been contaminated with BPA (Agarwal et al., 2016; Begum et al., 2020). Physical contact with thermal printed paper can result in the transfer BPA to human skin, which is detrimental to human health (Bernier and Vandenberg, 2017; Almeida et al., 2018).

In experiments with rodents, exposure to BPA during pregnancy and while nursing made the animals more anxious and prone to depression-like behaviors (Xu et al., 2012). Continuous exposure to BPA may result in the buildup of this hazardous chemical in tissues (Andra et al., 2016; Nayak et al., 2022). Previous studies have demonstrated that ingesting BPA through food can impair the colon’s normal permeability (Feng et al., 2019). Dietary BPA exposure also affects the morphological and biological barriers of the intestine, as well as its chemical environment (Feng et al., 2019). BPA is metabolized by the liver (Nachman et al., 2014). After absorption, certain enzymes, including uridine 5′diphospho-glucuronyl transferase (UGT) and sulfotransferases, convert unbound BPA into BPA-glucuronide and BPA-sulfate (Nachman et al., 2014). After detoxification, the body excretes BPA through urine, with a reported half-life of 2 h in blood (Neri et al., 2015). BPA can also be removed from the body through various routes, including sweat, feces, and bile (Genuis et al., 2012; Nayak et al., 2022). Due to increasing concerns about the safety of BPA, the industry has responded by adopting new, less studied BPA analogues that have similar polymer-forming properties (Kodila et al., 2023). Recent computational studies conducted in our lab have demonstrated that some BPA analogs have a higher binding affinity and toxic effect compared to BPA (Pathak and Kim, 2024). Despite avoiding them, humans can come into contact with BPA and its analog-based products due to their widespread availability (Wang et al., 2019; Sendra et al., 2023). Industry workers are exposed to it daily, and continuous exposure to this chemical is not safe (Konieczna et al., 2015; Vom Saal and Vandenberg, 2021). Therefore, multiple studies have recommended employing antioxidant treatment to reduce the toxic effects of BPA (Nayak et al., 2022). In addition, natural products are also suggested for the development of future therapeutics against BPA exposure (Sirasanagandla et al., 2022).

The interaction of bisphenols with ERRγ has been well documented (Liu et al., 2019; Ohore and Zhang, 2019). ERRγ is involved in the regulation of energy metabolism and mitochondrial function. Additionally, it directly or indirectly regulates numerous target genes (Misra et al., 2017). Mice without ERRγ do not survive their first week due to mitochondrial dysfunction. ERRγ is also vital for heart, muscle, and brain function (Alaynick et al., 2010; Misra et al., 2017). Furthermore, ERRγ is linked to various health issues, such as type 2 diabetes and alcoholic liver disease. In these conditions, inhibiting ERRγ through pharmacological means restores normal glucose levels and improves insulin sensitivity in mice (Kim et al., 2011; Kim et al., 2012; Kim et al., 2013; Misra et al., 2017). Therefore, the interactions of bisphenols with ERRγ interfere with its normal activity, leading to several dysfunctions (Babu et al., 2012; Nayak et al., 2022).

Previous studies on millets led to their recognition as smart food and highlighted the importance of their phytochemicals in human health, as well as their preventive role in pathologies such as cardiovascular diseases, diabetes, osteoporosis, cancer, and neurodegenerative disorders (Anitha et al., 2021; Kumar et al., 2021a; Lydia Pramitha et al., 2022; Kumar and Kotwal, 2023). Furthermore, millets aid in detoxification by removing toxins and free radicals while neutralizing enzymes within organs, thus preventing several health issues (Babele et al., 2022). The present study utilized millet-derived compounds to antagonize bisphenols-ERRγ interactions to mitigate the toxicity of BPA and its analogs.

Structure-based virtual screening is a well-recognized computational approach used to screen a database of small molecules against a molecular target of interest to investigate candidate compounds (Singh and Pathak, 2020; Pant et al., 2022). In the present study, virtual screening of 59 millet-derived phytochemicals was conducted, targeting amino acid residues of ERRγ that are involved in its interactions with bisphenols. During visualization of the protein-ligand complex, it was observed that higher molecular weight millet compounds, such as Tricin 7-rutinoside, adopted different poses, while lower molecular weight compounds, such as Setarin, showed similar poses to BPA. These compounds interacted with some of the residues where BPA binds to ERRγ, potentially antagonizing BPA’s binding to ERRγ. Our aim was to identify suitable millet-derived candidate compounds that can antagonize these interactions. To validate the findings, additional analyses were performed, including ADMET prediction, DFT calculations, molecular dynamics simulation, Gibbs free energy landscape analysis, and binding energy calculation (Pathak and Kim, 2024).

After conducting a rigorous assessment of compounds derived from millets, which were evaluated for their ability to antagonize bisphenols-ERRγ interactions, the ten most promising compounds were selected for detailed evaluation using a range of computational techniques (Maia et al., 2020). Subsequently, we examined the ADMET profile of these top ten compounds obtained from millets (Pires et al., 2015; Daina et al., 2017). It is worth highlighting that while a few of these leading compounds did not follow Lipinski’s Rule of 5, they still have significant potential as natural agents capable of antagonizing bisphenols-ERRγ interactions (Lipinski et al., 2001). We must acknowledge that numerous drug molecules currently in use have been approved despite not conforming to Lipinski’s Rule of 5 (Hartung et al., 2023). The DFT calculation analysis suggested that the chemical reactivity of the top five selected millet-derived compounds is comparable, allowing for favorable interactions with ERRγ (Bochevarov et al., 2013). Therefore, these identified molecules can be considered for further evaluation to determine their practical applicability.

Furthermore, we used an advanced computational method known as molecular dynamics simulation to evaluate the top five docked complexes: ERRγ-Tricin 7-rutinoside, ERRγ-Tricin 7-glucoside, ERRγ-Glucotricin, ERRγ-Kaempferol, and ERRγ-Setarin. This approach allowedus examine the interactions between these millet-derived compounds and ERRγ amino acids, where bisphenols are involved (Pant et al., 2022). By analyzing the RMSD plot, we found that all systems reached a stable state, indicating significant interactions with ERRγ. We also used other metrics such as RMSF, Rg, SASA, H-bonds, PCA, and FEL to investigate the stability of these complexes. The results showed that the millet-derived compounds formed robust and stable bonds with ERRγ, which is important for counteracting bisphenols-ERRγ interactions. This is a promising finding for potential medical applications (Priya et al., 2023).

Additionally, we investigated the binding ability of millet-derived compounds to ERRγ. We used a method called MM-PBSA to determine the strength of binding (Kuhn et al., 2005; Kumari et al., 2014). This approach provides insights into the potency of these compounds in their interactions with ERRγ, which is crucial for lead identification (Kuhn et al., 2005). The binding energy of Tricin 7-rutinoside, Tricin 7-glucoside, Glucotricin, Kaempferol, and Setarin were calculated. A negative binding energy indicates a strong binding between a ligand and receptor, signifying a favorable interaction. On the other hand, a high binding energy suggests a weaker interaction (Wan et al., 2020). Our analysis using MM-PBSA and residual binding energy demonstrated the stability of the complexes formed between millet-derived compounds and ERRγ. This finding highlights Glucotricin as a promising lead compound. However, other compounds such as Tricin 7-rutinoside, Tricin 7-glucoside, Kaempferol, and Setarin also show potential for further development and for antagonizing the interaction between bisphenols and ERRγ, which is important for future therapeutic advancements (Sirasanagandla et al., 2022; Priya et al., 2023).

Computer-assisted drug discovery can accelerate the process of identifying potential compounds by facilitating the analysis of plant-derived compounds against specific drug targets (Pathak et al., 2018; Pathak et al., 2020). This method reduces the time and financial resources required for experiments and enhances research outcomes (Pathak et al., 2020; Singh and Pathak, 2020). It is worth nothing that many of the medications available today are derived from plants or natural sources (Singh et al., 2022). Scientific research has already demonstrated the ability of natural products to mitigate the toxicity of bisphenols (Sirasanagandla et al., 2022). Bisphenols are well-known toxic compounds, but millets have been safely consumed by humans for centuries. While whole millets offer health benefits due to the synergistic effects of their phytochemicals, individual millet-derived compounds may behave differently. However, these compounds could be used in combination if found effective. Therefore, the findings of this study can be applied to the development of therapeutics that counteract the harmful effects of bisphenols by antagonizing their interactions with ERRγ.

## 5 Conclusion

The widespread use of bisphenols in various industries has raised serious concerns for human health due to their well-documented effects as endocrine disruptors. Research has shown that these compounds can interfere with the normal activity of ERRγ, a vital factor in energy metabolism and other biological functions, increasing the risk of various diseases. Our prior study revealed that some BPA analogs exhibit a stronger binding to ERRγ compared to BPA, which motivated us to investigate potential approaches to neutralize their toxic effects by inhibiting their interactions with ERRγ. Based on the health benefits and detoxification properties of millets, we screened millet-derived compounds, selected the top ten, and predicted their ADMET. Furthermore, prioritize the top five compounds to evaluate their reactivity through DFT analysis. Subsequently, molecular dynamics simulations were conducted on docked complexes of the top five millet compounds with ERRγ to assess their binding, stability, and antagonistic nature over time. Additionally, we refined our selections by evaluating their binding energies using the MM-PBSA method. These compounds exhibited potential therapeutic properties that might mitigate the health risks associated with bisphenols. However, it is crucial to emphasize the need for further research including wet-lab experiments and clinical studies to validate their efficacy and safety. These compounds, which might be utilized either as single molecules or in combination, could potentially play a vital role in protecting human health.

## Data Availability

The original contributions presented in the study are included in the article/Supplementary Material, further inquiries can be directed to the corresponding author.
