# Supplementary material for: A computational assay for identifying millet-derived compounds that antagonize the interaction between bisphenols and estrogen-related receptor gamma
Source: Front Pharmacol. 2024 Oct 31;15:1435254. doi: 10.3389/fphar.2024.1435254 (PMC11560427; doi:10.3389/fphar.2024.1435254)
Supplement: Supplementary file 2 [file DataSheet1.docx]

**A computational assay for identifying millet-derived compounds that antagonize the interaction between bisphenols and estrogen-related receptor gamma**

Rajesh Kumar Pathak and Jun-Mo Kim*

Department of Animal Science and Technology, Chung-Ang University, Anseong-si, Gyeonggi-do 17546, Republic of Korea

***Correspondence author**

Email: [junmokim@cau.ac.kr](mailto:junmokim@cau.ac.kr)

**Supplementary Figures**

**
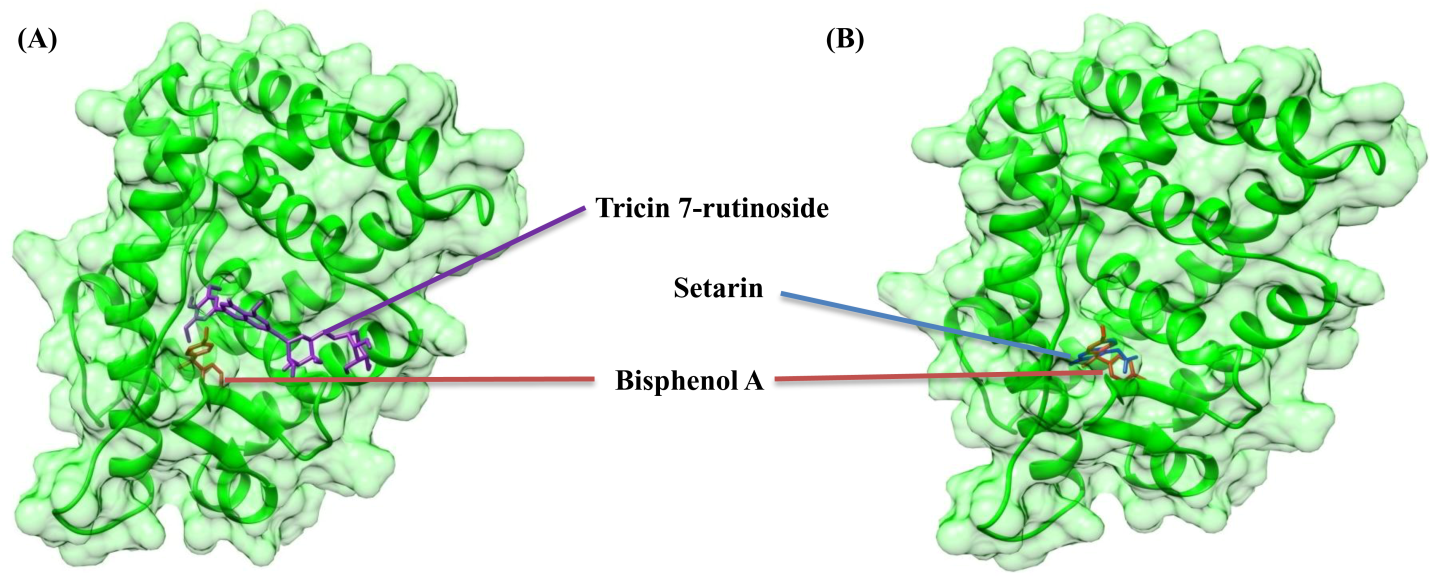
**

**Supplementary Figure S1.** Binding interactions of bisphenol A (BPA) and millet-derived compounds Tricin 7-rutinoside and Setarin with ERRγ.

**
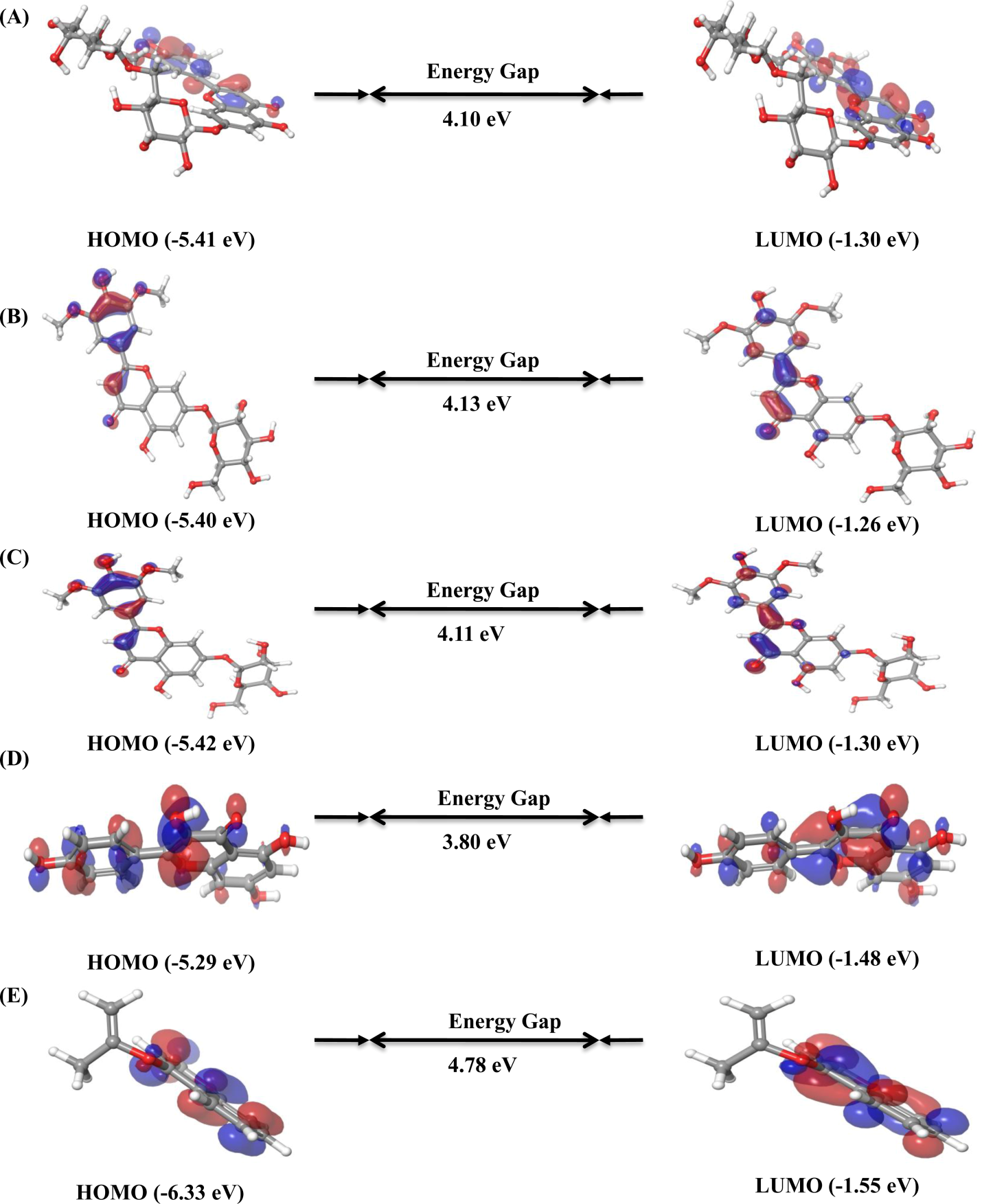
**

**Supplementary Figure S2.** Reactivity analysis based on DFT calculations, showing the visualization of HOMO and LUMO orbitals for millet-derived compounds: (A) Tricin 7-rutinoside, (B) Tricin 7-glucoside, (C) Glucotricin, (D) Kaempferol, (E) Setarin.

**
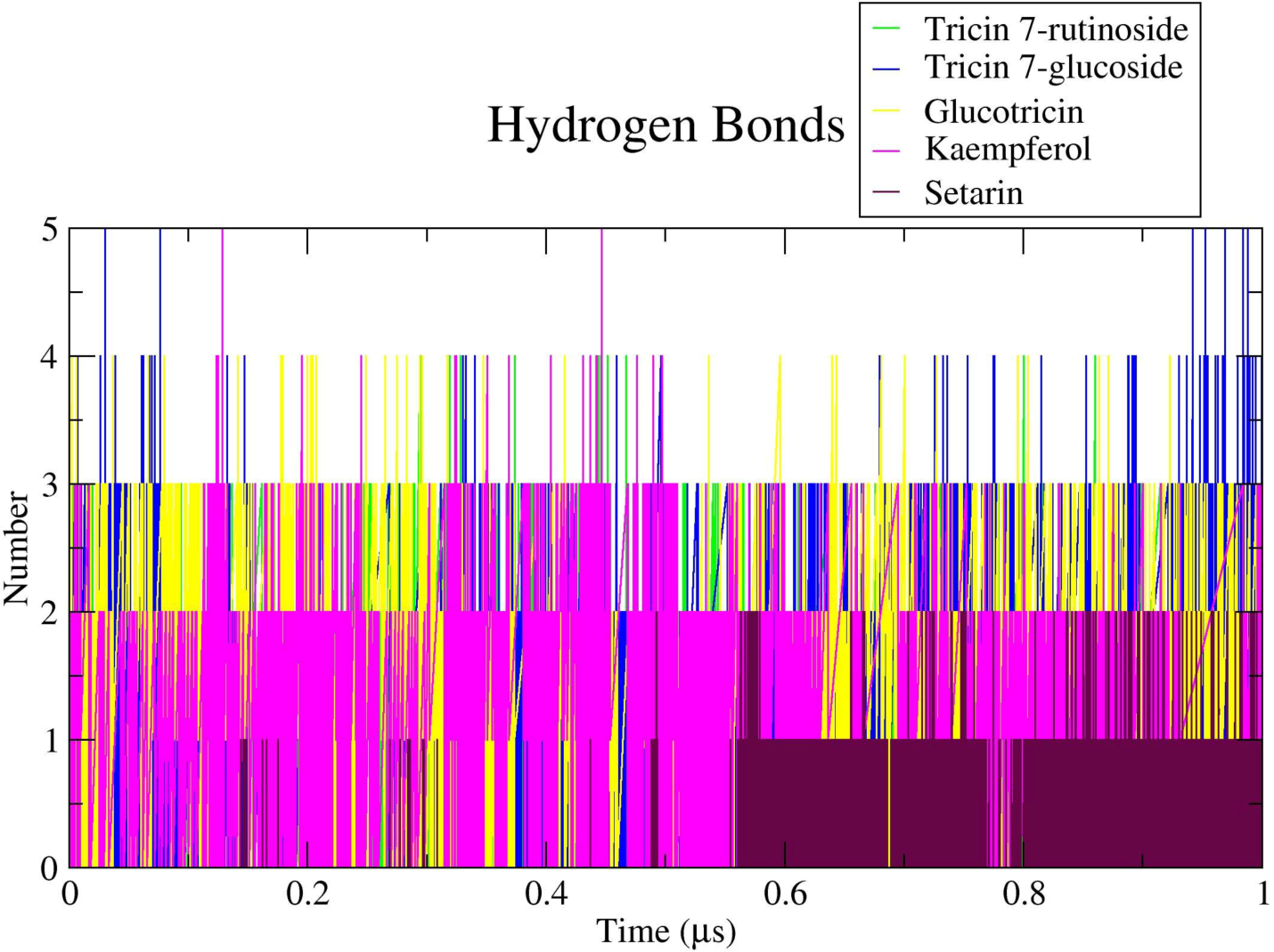
**

**Supplementary Figure S3.** Temporal progression of hydrogen bonds in ERRγ–millet-derived compound complexes as determined through molecular dynamics simulation. The x-axis represents simulation time (µs), while the y-axis represents the cumulative count of hydrogen bonds.


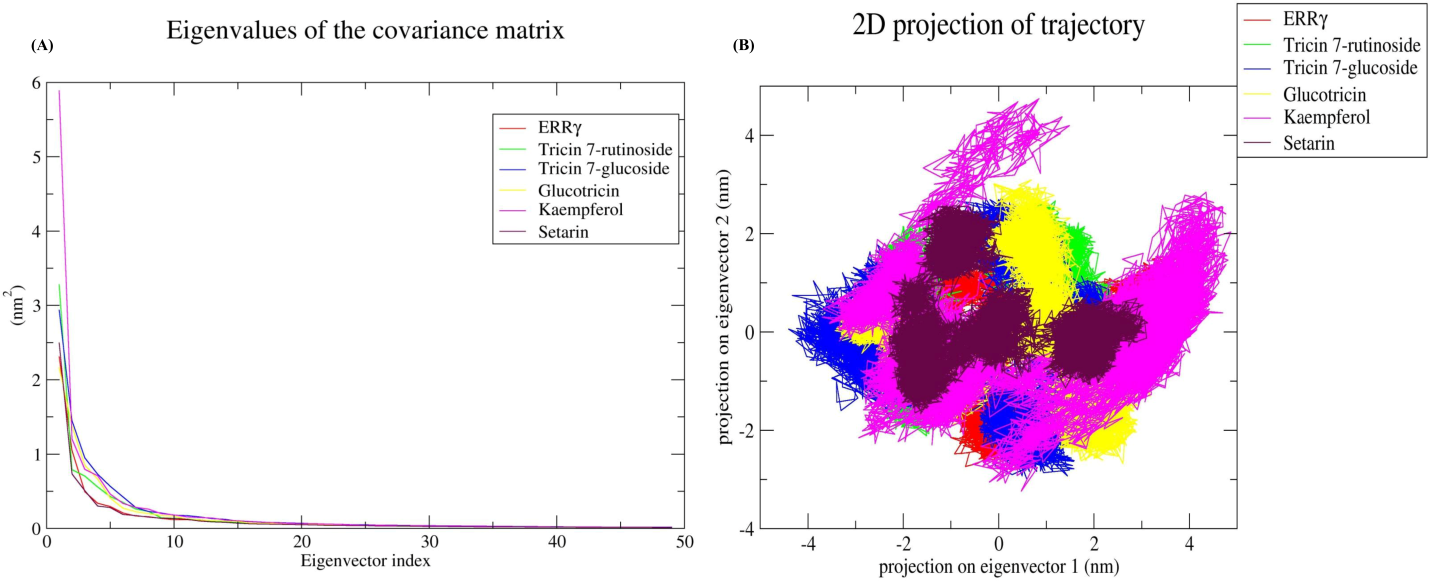


**Supplementary Figure S4.** Essential dynamics analysis through principal component analysis (PCA). **(A)** Eigenvalues obtained from simulations and used for PCA analysis, plotted against the first fifty eigenvectors. **(B)** The first two eigenvectors, revealing the spatial motion of ERRγ across all the systems.
